# Cases Cured by Dr. Hesse

**Published:** 1895-11

**Authors:** 


### CASES CURED.

By Dr. Hesse, Hamburg.

[From die Allg. Hom. Zeitung. Translated by A. M. McNeil, M. D., San Francisco, Cal.]

Mr. W., twenty-six years old, has suffered for thirteen weeks from oppression of the stomach which *was caused by violent anger*. Better when walking, and inclined to be better rather than worse after eating; *vertigo when walking in the open air, appetite bad. Physical restlessness.*

September 7th, 1892.—Sepia$^{30}$, to be given for three evenings.

September 10th.—Better after each powder. Appetite good, vertigo and restlessness improved. He did not consider it necessary to continue further treatment.

I have very frequently found that in chronic gastric affections after anger Sepia indicated; after cold food, Lycopodium.

L., a man of middle age, consulted me on account of a peculiar nervous vertigo, which is worse when he has time to think about it. *Aggravation when standing long;* in the heat of summer amelioration when busy, particularly in the open air. He feels as if drunk, aggravation morning and evening, easily oppressed in a room, he cannot endure sitting long.

He formerly had asthma, aggravated in the east wind.

April 4th, 1892.—Sepia$^{30}$, a dose every third evening.

April 27th.—His condition so much improved that he inquired whether it was necessary to continue treatment.

With Sepia often slight hints must suffice in selecting the remedy, as the restlessness when sitting, oppression in a room, and aggravation during idleness. So also in the following case:

A young lady, now about twenty years old, has had caries of both ankles since she was four. Five years ago she came under my care, when she had been confined to a wheel-chair for eleven years.

Both feet had lost their original shape, and were deformed lumps with eight or ten openings out of which more or less pus discharged.

Her general condition had always been good, and neither from her present or past state could I obtain anything in which to select a remedy. I had to grope about among the antipsorics, always a disagreeable thing to do, and thus for the first three years of my treatment I obtained no improvement. In the spring regularly there appeared new swelling and new openings, while the old fistules continued.

The incidental remark of my patient that she was very apt to become too warm in the room (even when it was not uncomfortable for others) caused me to give her Sepia. Since then her feet improved. For two years she has taken the 30th and 200th in weekly doses. No new fistulas appeared, but her feet lessened in size gradually so as to look more like those of a human being. At present there is only one fistula on each foot with but little discharge. She wears quite presentable shoes, which must occasionally be changed on account of her feet becoming thinner. She can stand on them, and is increasing in her ability to walk. The wheeled-chair has been discarded, although she had used it almost fifteen years. She goes by the aid of one or two canes, with a well-grounded hope of dispensing with them. In brief, she is in a condition that neither she nor her parents ever expected her to reach. The favorable change must be ascribed to Sepia.

In caries of the bones Bœnninghausen gives to Sepia an important position.

Emil E., æt. seven, has had a cough for several weeks, which, for the last eight days, has shown the characteristics of genuine whooping cough. Frequent spasmodic attacks of cough, always with vomiting of food and preceded by pains in chest. He had, besides, *soreness* of the nose, and always lay naked.

July 4th, 1892.—Sulphur$^{30}$, three powders, one each evening.

July 11th considerable improvement. Paroxysms seldom and slight, without vomiting. He now has a crusty eruption on the right cheek, which occurs every three months.

I gave him Placebos, and his mother did not think further treatment necessary. I afterward learned that his cough soon disappeared entirely. There was nothing characteristic in the cough, and the constant lying naked and the sore nose decided my choice for Sulphur.

Sulphur has soreness of nose; ulcerations in the nose and chronic coryza all in the first rank, but many other remedies have also. More important is the constant lying bare; in children this is a strong indication for Sulphur. I never neglect in children to inquire for this symptom. However, we must ascertain that it occurs in winter, in a cold room. This is the intolerance of the warmth of the bed of Sulphur. Children can tolerate no bed-covering; always have their legs out however often they may be covered. If the bed-clothes are so fastened that they cannot get their legs out their sleep is not so sound. Many times we find that after Sulphur is given they will keep covered.

In adults we find an analagous symptom, viz.: burning of soles of feet or of the feet at night in bed, so that he must have them out of the bed-covers.

(My experience is that these symptoms of Sulphur are the most important belonging to this drug, so that I seldom or never cure with it unless these symptoms are present.—McN.)

